# Evaluation of Oral Health and Oral Health-Related Quality of Life in Children with Adenoid Hypertrophy

**DOI:** 10.3390/children12091206

**Published:** 2025-09-10

**Authors:** Münevver Çoruh Kılıç, Kenan Cantekin, Emre Haylaz, Fahrettin Kalabalık, Korhan Kılıç, Hasan Basri Bircan, Mihriban Güner

**Affiliations:** 1 Department of Pediatric Dentistry, Faculty of Dentistry, Biruni University, Istanbul 34015, Turkey; munevverk@biruni.edu.tr; 2 Department of Pediatric Dentistry, Faculty of Dentistry, Sakarya University, Sakarya 54100, Turkey; kenancantekin@sakarya.edu.tr (K.C.); mihribanguner@sakarya.edu.tr (M.G.); 3 Department of Oral and Maxillofacial Radiology, Faculty of Dentistry, Sakarya University, Sakarya 54100, Turkey; fahrettinkalabalik@sakarya.edu.tr; 4 Department of Otolaryngology, Faculty of Medicine, Atatürk University, Erzurum 25240, Turkey; korhan.kilic@atauni.edu.tr; 5 Department of Orthodontics, Faculty of Dentistry, Atatürk University, Erzurum 25240, Turkey; hasanbasri.bircan@ogr.atauni.edu.tr

**Keywords:** hypertrophy, child, quality of life, malocclusion, oral health

## Abstract

**Background/Objectives**: Adenoid hypertrophy (AH), one of the most common pathologies in children, is a major cause of mouth breathing. Mouth breathing causes dry mouth, which removes the beneficial effects of saliva necessary for oral health. Therefore, an oral microflora favorable to caries is promoted. The primary objective of this study is to evaluate the oral health of children diagnosed with AH between the ages of 3 and 14 and to determine the early childhood oral health impact scale. **Methods**: This descriptive study was conducted between November 2019 and November 2020, involving 16 boys and 14 girls diagnosed with AH at the Department of Otorhinolaryngology, Faculty of Medicine, Atatürk University. These children, diagnosed with adenoid hypertrophy, were referred to the Department of Pedodontics, Faculty of Dentistry, Ataturk University, for the evaluation of their oral health prior to surgery. Oral examinations were performed on the pediatric participants under dental unit light by the same pedodontist, and their demographic data and DMFT/dmft scores were recorded. Data for the Early Childhood Oral Health Impact Scale (ECOHIS-T) were obtained from volunteers with communication skills and their parents. SPSS 21.0 software was used in the statistical evaluation of the data. A Chi-square test was used to assess differences between groups. **Results**: The mean age of the children was 5.9 ± 2.6 years. There was no statistically significant difference between AH grades 2, 3, and 4 in terms of dentition periods, gender, and occlusion (*p* = 0.177, *p* = 0.495). The scores of the first nine and last four questions of the ECOHIS-T were found to be higher in children with grade 4 AH (*p* = 0.011, *p* = 0.043). The DMFT index was also higher in children with grade 4 AH (*p* = 0.010). **Conclusions**: Tooth decay is more prevalent in children with severe adenoid hypertrophy. This condition was also observed to negatively affect their quality of life. Regular check-ups and preventive care are needed to improve the quality of life of these children.

## 1. Introduction

Waldeyer’s Ring is formed by the tubal tonsils located around the eustachian tube, the tonsils palatina located in the tonsillar fossa, the tonsils lingual located at the root of the tongue, and the tonsils pharyngea (adenoid) located in the nasopharynx. Adenoid tissue (tonsilla pharyngea) forms the central part of Waldeyer’s Ring and is located between the nasopharynx and the soft palate [1,2,3].

The adenoid begins to develop in the early stages of the embryonic period, around the 12th to 14th weeks of gestation. When fully matured, it reaches a weight of approximately 1.5 to 3.3 g and has a shape resembling a rounded-edged quadrilateral [1,4]. It tends to shrink and regress after puberty, and undergoes involution in adulthood [5,6]. The tonsil and adenoid tissues constitute the body’s first line of defense against antigens acquired through the oral and nasal routes [7,8,9].

AH, defined as the non-physiological enlargement of the nasopharyngeal tonsils, is the most common cause of nasal congestion in childhood. In the clinic, lateral cephalometric radiographs are the most straightforward method to diagnose AH [5]. Although AH can occur at different ages in children, it is most commonly seen between the ages of 2 and 6, and then undergoes a regression process [10,11].

AH can negatively affect the quality of life in children in various ways. Nasal obstruction and mouth breathing reduce sleep quality, leading to respiratory problems such as snoring and obstructive sleep apnea. These conditions may result in daytime fatigue, difficulty concentrating, and behavioral problems due to poor sleep [12,13,14,15,16]. Additionally, behavioral changes such as decreased school performance, irritability, daytime sleepiness, and morning headaches may occur, along with growth-related issues like headaches, low weight gain, and short stature [17,18].

AH can give rise to serious problems such as difficulties in speech, smell, taste, swallowing, snoring, obstructive sleep apnea, mouth breathing and maxillofacial anomalies, cardiopulmonary pathologies, and chronic alveolar hypoventilation [7,10,11]. Mouth breathing caused by AH has various effects on dentofacial development in children. Malocclusion is more commonly observed in children who mouth breathe compared to those who breathe through their noses. In children who mouth breathe, tongue pressure decreases and tongue muscles tend to assume a lower position. This situation leads to crowding of the upper teeth and narrowing of the maxillary dental arch, while also contributing to the development of posterior crossbite. Additionally, the mandible undergoes backward rotation and there is excessive eruption of the posterior teeth, which increases the risk of open bite [19,20,21,22]. Children who breathe through their mouths typically exhibit characteristic facial features such as a protruding upper lip, inadequate lip seal, nasal widening, a high-arched palate, and increased lower anterior facial height [21,23,24,25]. In addition, mouth breathing due to AH causes dry mouth, thus diminishing the positive effects of saliva, which is essential for oral health. As a result, an oral microflora favorable to caries development is promoted. It has also been shown that it can cause halitosis and periodontal problems [7,26,27].

Early interdisciplinary collaboration and implementation of screening protocols between otolaryngologists and pediatric dentists are of great importance in cases of AH. Early diagnosis and intervention support the healthy progression of both respiratory functions and maxillofacial development, contributing to the prevention of malocclusion and other complications. Such an interdisciplinary approach not only improves patients’ quality of life but also helps prevent the development of orthodontic and functional problems in the long term.

In recent years, much attention has been focused on oral health-related quality of life (OHRQoL) among children. Several OHRQoL measures have been developed for use with child populations [28]. Pahel et al. [29] developed a short questionnaire called the Early Childhood Oral Health Impact Scale (ECOHIS) derived from the child oral health quality of life scale (COHQoL) for children in the three- to six-year-old age group. This study aims to evaluate the oral health of children diagnosed with AH between the ages of 3 and 14 and to assess the impact of oral health during early childhood.

## 2. Materials and Methods

### 2.1. Study Design and Ethical Approval

This study was conducted on 30 patients diagnosed with AH at the Department of Otorhinolaryngology, Faculty of Medicine, Atatürk University, and referred to the Department of Pediatric Dentistry, Faculty of Dentistry, Atatürk University, for preoperative evaluation of oral health, and 30 healthy volunteer children. Thirty children who applied to the Ataturk University Hospital Ear, Nose, and Throat Polyclinic were examined by the same otorhinolaryngologist. Flexible fiberoptic nasopharyngoscopy (FFNP) was used for direct visualization of adenoids. Pharyngeal tonsil hypertrophy was determined according to Brodsky and Koch criteria. Grade 2, grade 3, and grade 4 children requiring adenoidectomy were included in the study. Oral examination of all 60 children included in the study, including the control group, was performed by the same dentist using a mirror and probe under reflector light. Informed consent was obtained from the parents. This study was approved by the Erzurum Regional Training and Research Hospital Clinical Research Ethics Committee according to the Declaration of Helsinki (Approval number: 2021/05-96; 25 May 2021).

### 2.2. Sample Size and Criteria

**Figure 1:**
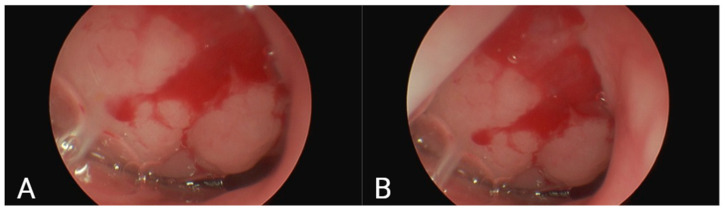
(**A**,**B**) Two different frames obtained from the FFNP of the same patient are shown.

The study group comprised children aged 3 to 14 years who presented with AH requiring adenoidectomy (grades 2, 3, or 4), exhibited mouth breathing, and had no systemic diseases. The control group consisted of age-matched children who demonstrated nasal breathing and had neither adenoid hypertrophy nor a history of adenoidectomy. AH is shown in the images obtained by FFNP (Figure 1).

Initially, a pilot study was conducted using G*Power software (Version 3.1.9.4, Franz Faul, Universität Kiel, Germany) to determine the appropriate sample size. For this pilot study, 10 subjects with adenoid hypertrophy and 10 age-matched controls without AH were randomly selected. According to the power analysis, a minimum of 28 subjects per group was required to achieve 95% power with an effect size of d = 0.988 and α = 0.05. To further enhance the power of the study, 30 subjects were included in each group.

Patients with a history of facial surgery, those diagnosed with dysmorphic or craniofacial syndromes, septal deviation, chronic diseases, acute upper respiratory tract infections, thumb-sucking habits, or those who had undergone or were undergoing orthodontic treatment were excluded from the study.

### 2.3. Data Collection

Demographic (age, gender, and parental education status), Early Childhood Oral Health Impact Scale (ECOHIS), and QoL data were assessed. The dentition period and dental occlusion were determined by intraoral examination, and the Decayed, Missing, and Filled Teeth (DMFT/dmft) index and ECOHIS-T were applied. In accordance with the requirements of the age group, the patient-reported outcomes were completed by the children’s parents.

#### 2.3.1. ECOHIS-T

The scale comprises 13 questions, each rated on a 5-point Likert scale. All collected data were recorded by the same pediatric dentist to ensure consistency. The first nine and the last four questions include child impact scale (CIS) and family impact scale (FIS), respectively. The CIS covers four domains, while the FIS covers two domains. The CIS comprises one question on symptoms, four questions on functional aspects, two questions on psychological processes, one question assessing self-perception, and one question evaluating social communication. The FIS consists of two questions regarding parenting-related distress and family function. Each question receives a score ranging from 0 to 5. Therefore, the total scores range from 0 to 52 (0 = 0 never; 1 = almost never; 2= occasionally; 3 = frequently; 4 = very frequently; 5 = I don’t know) (Table 1).

#### 2.3.2. DMFT Index

Participants’ decayed, missing, and filled teeth were identified, and the data were recorded using the DMFT/dmft index (DT: decayed permanent teeth, MT: missing permanent teeth, FT: filled permanent teeth; dt: decayed primary teeth, mt: missing primary teeth, ft: filled primary teeth) [30].

### 2.4. Statistical Analyses

Statistical analyses were performed using SPSS for Windows software (version 22.0; IBM Corp., Armonk, NY, USA, ABD). The intra-observer coefficient of variation for all assessments was 0.94. Normality tests were performed for all variables. Among children with adenoid hypertrophy, age, gender, parental education level, dentition period, occlusion type, DMFT/dmft scores, and ECOHIS-T results were compared using the Mann–Whitney U test. Correlations between DMFT/dmft scores and ECOHIS-T scores were assessed using Spearman’s rank correlation analysis. A *p*-value of <0.05 was considered statistically significant.

## 3. Results

The study included a total of 60 pediatric participants. The average age was 8.71 ± 3.50 years in the AH group and 8.71 ± 3.50 years in the control group. The overall mean age of the children in the study was 7.53 ± 2.641 years. There was no statistically significant difference in mean age between the study groups (*p* = 0.08). Of the children included in the study, 26 (43.3%) were females and 34 (56.7%) were males. There was no statistically significant difference in gender distribution between the groups (*p* = 0.177) (Table 2).

The total DMFT index of the study was 6.27 ± 2.641, 8.39 ± 4.58 in the AH group, and 3.86 ± 5.21 in the control group. The DMFT index was found to be higher in children with AH (*p* = 0.010). There was no statistically significant difference between AH grade 2, 3, 4 and the dentition periods and occlusion relationship (*p* = 0.25, *p* = 0.49) (Table 2).

Table 3 presents the OHRQoL data of all children included in the study. Comparison of ECOHIS scores between the groups revealed a statistically significant difference only in the children’s self-image and social interaction subscale scores (*p* = 0.048). The patient group exhibited a statistically significantly higher score compared to the control group. This finding indicates that children with AH experience a more negative quality of life in this specific subscale compared to the control group. In particular, it appears that social relationships and self-image are more adversely affected in children within the patient group. This suggests that AH is not merely a physical health issue but also a condition that significantly impacts children’s psychosocial development and overall quality of life. Therefore, the findings highlight that neglecting oral health in children can lead not only to clinical consequences but also to notable negative effects in social and emotional domains.

## 4. Discussion

AH, one of the most common causes of upper respiratory tract obstruction in children, often leads to mouth breathing instead of nasal breathing. Mouth breathing caused by AH results in dry mouth, thereby diminishing the beneficial effects of saliva, which plays a crucial role in maintaining oral health. Consequently, this condition promotes an oral microflora conducive to the development of dental caries [7,31,32]. When nasal breathing is replaced by mouth breathing, the moisturizing and antimicrobial effects of saliva are reduced. As a result, issues such as dryness, irritation, and inflammation of the oral mucosa may occur. This condition makes the oral mucosa more vulnerable to bacterial and fungal infections and can also lead to socially disturbing symptoms such as halitosis [21,33,34,35]. Bad breath, mucosal discomfort, and esthetic concerns can negatively impact children’s self-perception and social interactions. Additionally, due to mucosal sensitivity, functions such as eating and speaking may also be affected. All of these effects can reduce children’s OHRQoL [34,36]. Therefore, in children diagnosed with AH, it is of great importance to initiate preventive oral care practices at an early stage to prevent mucosal complications and to ensure multidisciplinary collaboration among relevant specialties.

Unlike the lateral cephalometric radiographs frequently used in other studies, this study preferred FFNL for the evaluation of AH [37,38]. FFNL offers high diagnostic accuracy due to its ability to visualize the adenoid tissue directly and in real time, and it is considered the gold standard method in the literature. In this regard, the use of FFNL provides more accurate, dynamic, and clinically meaningful information in the diagnosis of adenoid hypertrophy, setting this study apart from previous research [39,40].

In the study conducted by Farahzadi et al. [37], the prevalence of dental caries in children with adenoid hypertrophy was reported to be significantly higher compared to healthy children. In the study, the DMFT values were 4.10 ± 2.09 and 2.06 ± 0.97, and the dmft values were 3.52 ± 3.34 and 1.48 ± 1.24 in the case and control groups, respectively [37]. Ballıkaya et al. stated that mouth breathing is a risk factor for cariogenic microorganisms [26]. The outcomes of this research may also suggest that the risk of caries is increased in children with AD. These findings indicate that the risk of dental caries is increased in children with AH, with mouth breathing considered a significant contributing factor to this increase. In addition to the risk of caries, chronic mouth breathing and reduced salivary flow in AH may predispose children to mucosal complications such as inflammation and soft tissue irritation, as observed in similar contexts by Manuelli et al. [36]. Therefore, implementing preventive oral care practices and appropriate treatment strategies at an early stage in children diagnosed with AH is crucial for preventing the development of dental caries.

Numerous studies have shown that mouth breathing associated with AH leads to dentofacial changes, and most of these anomalies are reversible after treatment [7,26,27,41]. However, no significant difference was found between the groups in terms of Class I occlusion in this study. This may be attributed to the selection of control group children from a population predisposed to dental caries, as they presented with pain or other dental complaints and were referred for orthodontic evaluation.

Oral hygiene problems, which are among major public health issues, affect an individual’s quality of life socially, economically, and psychologically. When ECOHIS scores were compared between the groups, no statistically significant differences were observed in terms of child symptoms, functional limitations, psychological impact, or family distress and functioning. A significant difference was found only in the domains of children’s self-image and social interaction. Inönü-Sakallı et al. [7] attributed this situation to the higher self-image and social interaction scores in the patient group compared to the control group, which may be due to dentofacial changes and halitosis. AH has not been shown to have a negative impact on children’s OHRQoL. Although AH may be a risk factor for dental caries, maintaining proper oral hygiene can help prevent the development of caries in affected children.

In cases of AH, early collaboration between otolaryngologists and pediatric dentists, along with the implementation of screening protocols, is extremely important. Early diagnosis and treatment support the healthy development of respiratory functions and contribute to the proper progression of the maxillofacial structure, thereby helping to prevent malocclusion and other potential complications. This interdisciplinary approach not only enhances patients’ quality of life but also helps prevent the emergence of orthodontic and functional problems in the long term.

One of the main limitations of our study is that, as a pilot study with a limited sample size, it was not possible to compare the outcomes of the post-adenoidectomy treatment group with those of the patient and control groups. Additionally, the investigation of other factors influencing caries risk was beyond the scope of this study and is left for future research. Furthermore, Grade 2, 3, and 4 groups could not be evaluated separately.

## 5. Conclusions

This study aimed to evaluate the oral health of children aged 3 to 14 diagnosed with AH and to examine the impact of oral health during early childhood. Although AH does not appear to have a negative effect on oral health-related quality of life, dental caries are more prevalent in affected children. Therefore, consulting a pediatric dentistry specialist is necessary for oral rehabilitation, identifying risk factors, and providing education and support to both the child and their family. Preventive strategies, including the use of remineralization agents, are important to prevent the development of new carious lesions. Effective treatment of children with AH should be carried out through a multidisciplinary approach involving collaboration among otorhinolaryngologists, pediatric dentists, and orthodontists.

## Figures and Tables

**Table 1:** Descriptive statistics of the ECOHIS item responses.

Response Options: 0 = Never; 1 = Almost Never; 2 = Occasionally; 3 = Frequently; 4 = Very Frequently; 5 = I Don’t Know
**Child Impact Section**
**1.** Has your child ever had pain in the teeth, mouth, or jaws (bones of the mouth)?
**2.** Has your child ever had difficulty drinking hot or cold drinks due to problems with teeth or dental treatments?
**3.** Has your child ever had trouble eating certain foods due to problems with teeth or dental treatments?
**4.** Has your child ever had difficulty pronouncing any words due to problems with teeth or dental treatments?
**5.** Has your child ever missed daycare, kindergarten, or school due to problems with teeth or dental treatments?
**6.** Has your child ever had trouble sleeping due to problems with teeth or dental treatments?
**7.** Has your child ever been irritated by problems with teeth or dental treatments?
**8.** Has your child ever avoided smiling or laughing due to problems with teeth or dental treatments?
**9.** Has your child ever avoided talking due to problems with teeth or dental treatments?
**Family Impact Section**
**10.** Have you or someone else in the family ever been upset because of problems with your child’s teeth or dental treatments?
**11.** Have you or someone else in the family ever felt guilty because of problems with your child’s teeth or dental treatments?
**12.** Have you or someone else in the family missed work due to problems with your child’s teeth or dental treatments?
**13.** Has your child ever had problems with his teeth or had dental treatments that have had a financial impact on your family?

**Table 2:** Evaluation of demographic parameters, DMFT index, dentition period, and dental occlusion.

	Gender (Girls–Boys)	Age (Avg. ± SS)	DMFT (Avg. ± SS)	N, DP% D-K-S	N, DO% Class 1
Control Group	30 (14–16)	7.35 ± 4.85	3.86 ± 5.21	6-9-0 40%, 60%, 0	8, 53%, 3
Adenoid Hypertrophy Grade (2,3,4)	30 (12–18)	8.71 ± 3.50	8.39 ± 4.58	5-7-3 33.3%, 46.6%, 20%	5, 33%, 3
Total	60 (26–34)	7.53 ± 2.641	6.27 ± 5.21	11-16-3 36.6%, 53.3%, 10%	13, 43%, 3
*p*	0.177	0.08	0.010 *	0.25	0.49

Note: * *p* < 0.05, The Mann–Whitney U test was performed, and statistically significant *p*-values are indicated in bold. DMFT: Decayed, Missing, and Filled Teeth Index; DP: dentition period; D: permanent; M: mixed; P: primary; DO: dental occlusion.

**Table 3:** Assessment of OHRQoL-ECOHIS.

	Group	(Avg. ± SS)	*p* Value
Child Impact Domain			
Symptoms in Children	AHG	0.97 ± 1.32	0.948
	CG	1.08 ± 1.24	
	Total	1.03 ± 1.42	
Child Functional Status	AHG	2.07 ± 3.23	0.789
	CG	2.46 ± 3.64	
	Total	2.42 ± 3.55	
Child Psychology	AHG	0.52 ± 1.27	0.807
	CG	0.65 ± 1.57	
	Total	0.57 ± 1.45	
Child’s Self-Perception and Social Interaction	AHG	1.93 ± 1.78	0.048 *
	CG	0.98 ± 1.47	
	Total	1.43 ± 1.67	
Total Child Score	AHG	5.37 ± 6.72 5.0 ± 6.87	0.520
	CG		
	Total		
Family Impact Section			
Parental Distress	AHG	0.68 ± 1.54	0.348
	CG	1.24 ± 2.12	
	Total	1.00 ± 1.82	
Family Function	AHG	0.50 ± 0.92	0.134
	CG	0.76 ± 1.52	
	Total	0.55 ± 1.62	
Total Parent Score	AHG	0.89 ± 2.23	0.560
	CG	1.93 ± 3.49	
	Total	1.53 ± 3.09	
Total ECOHIS Score	AHG	6.57 ± 8.62	0.690
	CG	6.81 ± 9.94	
	Total	6.59 ± 9.63	

Note: * *p* < 0.05, AHG: adenoid hypertrophy group (Grade 2 + Grade 3 + Grade 4), CG: control group.

## Data Availability

Most of the data generated or analyzed are included in the article. The remaining datasets used and/or analyzed during the current study are available from the corresponding author upon request.
